# Dispersed Conducting Polymer Nanocomposites with Glucose Oxidase and Gold Nanoparticles for the Design of Enzymatic Glucose Biosensors

**DOI:** 10.3390/polym13132173

**Published:** 2021-06-30

**Authors:** Natalija German, Almira Ramanaviciene, Arunas Ramanavicius

**Affiliations:** 1 Department of Immunology, State Research Institute Centre for Innovative Medicine, Santariskiu 5, LT-08406 Vilnius, Lithuania; natalija.german@imcentras.lt (N.G.); almira.ramanaviciene@chf.vu.lt (A.R.); 2 NanoTechnas—Center of Nanotechnology and Materials Science, Faculty of Chemistry and Geosciences, Vilnius University, LT-03225 Vilnius, Lithuania; 3 Department of Physical Chemistry, Faculty of Chemistry and Geosciences, Vilnius University, Naugarduko 24, LT-03225 Vilnius, Lithuania; 4 Division of Materials Science and Electronics, State Scientific Research Institute Center for Physical Sciences and Technology, Savanorių ave. 231, LT-02300 Vilnius, Lithuania

**Keywords:** conducting polymers, glucose biosensor, glucose oxidase, gold nanoparticles, graphite rod electrode, interference by ascorbic and uric acids, polyaniline, polypyrrole, amperometry, bioelectrochemistry

## Abstract

Biosensors for the determination of glucose concentration have a great significance in clinical diagnosis, and in the food and pharmaceutics industries. In this research, short-chain polyaniline (PANI) and polypyrrole (Ppy)-based nanocomposites with glucose oxidase (GOx) and 6 nm diameter AuNPs (AuNPs_(6 nm)_) were deposited on the graphite rod (GR) electrode followed by the immobilization of GOx. Optimal conditions for the modification of GR electrodes by conducting polymer-based nanocomposites and GOx were elaborated. The electrodes were investigated by cyclic voltammetry and constant potential amperometry in the presence of the redox mediator phenazine methosulfate (PMS). The improved enzymatic biosensors based on GR/PANI-AuNPs_(6 nm)_-GOx/GOx and GR/Ppy-AuNPs_(6 nm)_-GOx/GOx electrodes were characterized by high sensitivity (65.4 and 55.4 μA mM^−1^ cm^−2^), low limit of detection (0.070 and 0.071 mmol L^−1^), wide linear range (up to 16.5 mmol L^−1^), good repeatability (RSD 4.67 and 5.89%), and appropriate stability (half-life period (τ_1/2_) was 22 and 17 days, respectively). The excellent anti-interference ability to ascorbic and uric acids and successful practical application for glucose determination in serum samples was presented for GR/PANI-AuNPs_(6 nm)_-GOx/GOx electrode.

## 1. Introduction

The design and wide application of electrochemical biosensors in nanoscience, nanotechnology, medicine, environmental, and food monitoring has significantly intensified during the past decade [1,2,3,4,5,6]. The advanced technology has enabled the construction of highly sensitive, selective, customizable, and portable sensors for the determination of glucose [3,4,5,6,7,8,9]. The development of glucose biosensors has great significance in the diagnosis and control of diabetes mellitus, which is considered a worldwide public health problem, which increases the risk of heart disease, kidney failure, blindness, postoperative and wound infections [2,3,4,5,10,11]. Diabetes is diagnosed in seven million people every year and globally one person is killed every 10 s due to this disease [2]. Glucose biosensors have been become widespread due to the high speed, accuracy, and simplicity of analyte’s level monitoring in the blood [2,3,6,11,12], saliva [4], tears and sweat [10,13].

Recently, enzymes have been used for the design of enzymatic biofuel cells [14,15,16,17,18] and biosensors [8,9,13,16,17,18,19,20]. During electrochemical processes, glucose oxidase (GOx) does not directly transfer electrons towards conventional electrode materials, because a thick protein layer surrounds the active site of GOx based on the flavin adenine dinucleotide (FAD) redox center, and this protein layer is forming the intrinsic barrier to charge transfer from the active site [10,15,21]. Therefore, in many GOx-related researches, charge transfer is accelerated by soluble redox mediators (e.g., phenazine methosulfate (PMS)) [8,10]. In the presence of oxygen in the solution, GOx immobilized on graphite electrodes catalyzes the oxidation of glucose into gluconolactone following hydrolysis to gluconic acid and provides the selectivity for the electrochemical sensor [2,14,21,22,23,24,25]. Usually the oxidation of glucose is proceeded at the potential, which is slightly more positive than the redox potential (−0.44 V) of FAD cofactor located in the active site of GOx, and this redox potential is sufficient for the reduction of dissolved oxygen [22,23,26]. The development of miniaturized enzymatic biosensors with the size ranging from several millimeters down to a few micrometers requires the combination of knowledge in electrochemistry, material science, polymer synthesis, enzymology and biological chemistry [4,20].

Major fundamental and nanotechnological advances have opened new horizons for the application of nanomaterials [10,20] and hybrid composites (carbon nanotubes [25], carbon nanotubes/graphene oxide [9], chitosan/gold nanoparticles [27]) in bioanalytical chemistry [10]. Compositions of nanomaterials (Ag, Au, MnO_2_, SiO_2_, ZnO, ZrO_2_, and many others), which are characterized by variable sizes and shapes, have been considered as unique materials for the immobilization of enzymes and have been used for the design of electrochemical biosensors [1,22,23,24,27,28,29]. The nature of the interaction between working electrodes, gold nanoparticles (AuNPs) and proteins has not been fully investigated and the influence of surface to volume ratio, high energy, and very active AuNPs as electron transfer between a redox protein and the surface of an electrode occurs in several ways [1,30]. Firstly, AuNPs are able to strongly interact with some parts of protein molecules: in such a way, proteins become immobilized on the surface of AuNPs. If the redox active sites of proteins are at a sufficiently low distance from AuNPs surface, then the electron transfer can proceed much easier [30,31]. AuNPs are characterized by high conductivity and biocompatibility, they are able to improve the sensitivity and stability of biosensors by catalyzing the oxidation of H_2_O_2_ and the facilitation of accepted electron transfer towards the electrode [7,10,22]. GOx is able to catalyze the reduction of AuCl_4_^−^ ions and form of metallic AuNPs, the size of which depends on the kinetics of the nucleation reaction [26].

*π*–*π* conjugated polymers (including polyaniline (PANI) and polypyrrole (Ppy)), which can be synthesized via chemical or electrochemical oxidation of monomers, are used for the development of rechargeable batteries, smart windows, light emitting diodes, transistors, sensors and etc. [32]. These conjugated polymers also are characterized by excellent electrical properties, therefore, in some particular cases they may act as redox mediators and facilitate the transfer of electrons to the electrode [1,16,17,33]. The formation of charge carriers such as polarons, bipolarons, and solitons is leaded due to electrons addition or extraction from the delocalized *π*-boned polymer backbone [32,34]. The colors of polymers depend on their redox states, e.g., PANI changes the color from yellow to green, blue, and violet [34], and Ppy exists in intensive black color due to the delocalized *π*-electron system of the doped polymer [35]. The conductivity of *π*–*π* conjugated polymers can be improved by electrochemical doping to induce an insulator-to-conductor transition in these polymers [34], and both *n*-type and *p*-type charge carriers can be involved in charge transfer within *π*–*π* conjugated polymers. PANI is electrically conducting and electroactive in protonated form at low pH values, but at pH 3 or 4 it is insulating and electrochemically inactive [32]. During the enzymatic formation of *π*–*π* conjugated polymers, the monomer is ‘attacked’ by a radical cation induced by H_2_O_2_ and this process is leading towards the polymerization reaction [32,36]. The *π*–*π* conjugated structures formed between the redox center of the enzyme and the electrode increase the efficiency of the charge transfer in this structure [10,16,17,33].

PANI and Ppy were combined with electroactive AuNPs and GOx, and were integrated as a structure suitable for the development of biosensors dedicated for the monitoring of glucose [1,29]. The hydrophilic functional groups (e.g., −NH_2_) of proteins paved the way to the interaction of AuNPs with these proteins [30,37]. The biosensors based on *π*–*π* conjugated polymer nanocomposites presented the best sensing performance due to the effective ‘contact’ between the enzyme and electrode. Additionally, such types of biosensors are more resistant to some interfering materials [4,29,34]. The developed glucose biosensor based on graphite rod (GR) electrode modified by an overoxidized polypyrrole (OOPpy_(300 s)_) film, nano Au and GOx and operating in the presence of redox mediator was characterized by the sensitivity of 3.07 μA mM^−1^ cm^−2^ [29]. The glucose biosensor based on GR electrode modified with electrochemically deposited 13 nm AuNPs, immobilized GOx and formed Ppy film exhibited sensitivity to glucose of 21.7 μA mM^−1^ cm^−2^ in the presence of redox mediator [38]. Biosensor based on GR electrode modified by long-chain Ppy nanocomposites containing GOx and AuCl_4_^−^ ions was characterized by a sensitivity of 4.31 mA mM^−1^ cm^−2^ and narrow linear range (up to 0.70 mmol L^−1^) in the presence of redox mediator [39].

The main aim of the presented study was to improve the analytical characteristics of amperometric glucose biosensors based on conducting polymer nanocomposites with embedded GOx and 6 nm diameter AuNPs (AuNPs _(6 nm)_). For this reason, short chain polymer nanocomposites (PANI-AuNPs_(6 nm)_-GOx and Ppy-AuNPs_(6 nm)_-GOx) were prepared, dispersing *π*–*π* conjugated polymer-based nanocomposites in the solution by ultrasound. An additional layer of GOx was formed over GR electrode pre-modified with dispersed conducting polymer nanocomposites.

## 2. Materials and Methods

### 2.1. Materials

Glucose oxidase (EC 1.1.3.4, type VII, from *Aspergillus niger*, 201 units mg^−1^ protein) and D-(+)-glucose, D(−)-fructose, D(+)-mannose, D(+)-galactose, D(+)-xylose, D(+)-saccharose were obtained from Fluka (Buchs, Switzerland) and Carl Roth GmbH+Co.KG (Karlsruhe, Germany). Tetrachloroauric acid and tannic acid were purchased from Alfa Aesar GmbH&Co KG and Carl Roth GmbH + Co (Karlsruhe, Germany), sodium citrate—from Penta (Praha, Czech Republic). 0.05 mol L^−1^ solution of sodium acetate (SA) buffer with 0.1 mol L^−1^ potassium chloride (KCl) was prepared from sodium acetate trihydrate and potassium chloride, whose were obtained from Reanal (Budapest, Hungary) and Lachema (Neratovice, Czech Republic). Aniline and sodium hydroxide were purchased from Merck KGaA (Darmstadt, Germany), pyrrole—from Acros Organics (New Jersey, NJ, USA), graphite rod (GR, Ø = 3 mm) and hydrochloric acid—from Sigma-Aldrich (Saint Louis, MO, USA), *α* alumina powder (Al_2_O_3_, 0.3 μm, Type N)—from Electron Microscopy Sciences (Hatfield, MA, USA). The solution of 25% glutaraldehyde was obtained from Fluka Chemie GmbH (Buchs, Switzerland), phenazine methosulfate, L-ascorbic acid (AA) and uric acid (UA)—from AppliChem GmbH (Darmstadt, Germany). 2.3 × 10^16^ particles L^−1^ of AuNPs_(6 nm)_ were prepared as was described in previous research [28].

### 2.2. The Polymerization, Separation and Dispersion of Polymer-Based Nanocomposites

PANI-GOx, Ppy-GOx, PANI-AuNPs_(6 nm)_-GOx and Ppy-AuNPs_(6 nm)_-GOx nanocomposites were synthesized for 2 days and separated according to previously described methodology [39,40,41]. PANI-AuNPs_(6 nm)_-GOx and Ppy-AuNPs_(6 nm)_-GOx were tending to agglomerate according to previously reported results [39,42]. In the presence of GOx and AuNPs_(6 nm)_ aniline and pyrrole were polymerized into the spherical particles. In the presence of pyrrole, nanocomposites were formed layered structure.

The separation of polymer nanocomposites from the polymerization solution was performed at 14.6 × 10^3^ g for 8 min using IEC CL31R Multispeed centrifuge (ZI Aze Bellitournt, France). After then, the collected polymer-based nanocomposites were dispersed in 40 μL of SA buffer, pH 6.0, using the ultrasonic bath (Bandelin electronic, Germany) to achieve short chain polymers.

### 2.3. The Preparation of Graphite Rod Electrode and Modification by Polymer-Based Nanocomposites

Working electrode — graphite rod (surface area 0.071 cm^2^) was cut, polished using fine emery paper and by humid Al_2_O_3_, washed, and dried at +20 ± 2 °C. Then, GR was placed into a silicone tube. 0.43 μL mm^−2^ of dispersed polymer nanocomposites were deposited on CR electrode. Then 3 μL of 25 mg mL^−1^ GOx were immobilized on a graphite rod electrode modified by polymer nanocomposite. After the evaporation of water at room temperature, the prepared working electrode was stored for 15 min in a closed vessel over a 25% solution of glutaraldehyde at +20 ± 2 °C, which covalently cross-linked the enzyme adsorbed on the polymer nanocomposites deposited on the GR [43].

### 2.4. Application of Glucose Biosensor Based on GR/PANI-AuNPs_(6 nm)_-GOx/GOx Electrode for Glucose Detection in the Serum Sample

The selectivity tests of glucose biosensor to interfering and electroactive species and the electrochemical determination of glucose concentration in diluted (1:10) serum sample using GR/PANI-AuNPs_(6 nm)_-GOx/GOx electrode was performed according to a methodology presented previously [39]. The influence of other sugars on the current response generated by biosensor was tested in serum sample containing 10.0 mmol L^−1^ of glucose in the absence and presence of 1.00 mmol L^−1^ fructose, mannose, galactose, xylose, and saccharose. To investigate the effect of AA and UA on the GR/PANI-AuNPs_(6 nm)_-GOx/GOx electrode, serum samples with 10.0 mmol L^−1^ of glucose; 10.0 mmol L^−1^ of glucose and 0.01, 0.10 or 0.20 mmol L^−1^ of AA; 10.0 mmol L^−1^ of glucose and 0.01, 0.05 or 0.10 mmol L^−1^ of UA were used.

### 2.5. Electrochemical and Statistical Evaluations of GR/PANI-GOx/GOx, GR/Ppy-GOx/GOx, GR/PANI-AuNPs_(6 nm)_-GOx/GOx or GR/Ppy-AuNPs_(6 nm)_-GOx/GOx Electrodes

Electrochemical measurements were performed by the computerized potentiostat PGSTAT 302/Autolab from EcoChemie (Utrecht, Netherlands) with GPES 4.9 software. GR/PANI-GOx/GOx, GR/Ppy-GOx/GOx, GR/PANI-AuNPs_(6 nm)_-GOx/GOx or GR/Ppy-AuNPs_(6 nm)_-GOx/GOx electrodes were switched into electrochemical cell a working electrodes, 2 cm^2^ platinum electrode was used as a counter electrode and Ag/AgCl_(3 mol L^−1^_ _KCl)_ from Metrhom (Herisau, Switzerland) — as a reference electrode.

The registration of glucose was performed at +0.30 V vs. Ag/AgCl_(3 mol L^−1^_ _KCl)_ using amperometry in stirred (1400 rpm) by a magnetic stirrer IKA from Werke GmbH & Co. KG (Staufen, Germany) solution of 0.05 mol L^−1^ SA buffer, pH 6.0, containing 0.1 mol L^−1^ KCl and in the presence of 6 mmol L^−1^ PMS as soluble redox mediator. Cyclic voltammograms were registered at potential diapason from −0.60 to +0.80 V vs. Ag/AgCl_(3 mol L^−1^_ _KCl)_ at a potential sweep rate of 0.100 V s^−1^ in an unstirred solution of 0.05 mol L^−1^ SA buffer, pH 6.0, containing 0.1 mol L^−1^ KCl and in the presence 6 mmol L^−1^ of PMS.

The sample of the serum was diluted (1:10) in 0.05 mol L^−1^ SA, pH 6.0, with 0.1 mol L^−1^ KCl and centrifuged within 8 min (14.6 × 10^3^ g). All investigations were performed with biosensors based on GR/PANI-AuNPs_(6 nm)_-GOx/GOx electrode. The electrochemical measurements were applied in 10× diluted serum sample with 10.0 mmol L^−1^ of glucose before and after the addition of 1.00 mmol L^−1^ fructose, mannose, galactose, xylose and saccharose. To evaluate the influence of ascorbic and uric acids on the developed biosensor, the analytical signals of glucose were registered in the solutions of 10× diluted serum sample with: 10.0 mmol L^−1^ of glucose; 10.0 mmol L^−1^ of glucose and 0.01, 0.10 and 0.20 mmol L^−1^ of AA; 10.0 mmol L^−1^ of glucose and 0.01, 0.05 and 0.10 mmol L^−1^ of UA.

The parameters of Michaelis–Menten kinetics (the difference of maximal current generated during the enzymatic reaction (Δ*I*_max_) and the apparent Michaelis constant (*K*_M_)) and the limit of detection (LOD) were estimated by SigmaPlot software 12.5.

**Figure 1:**
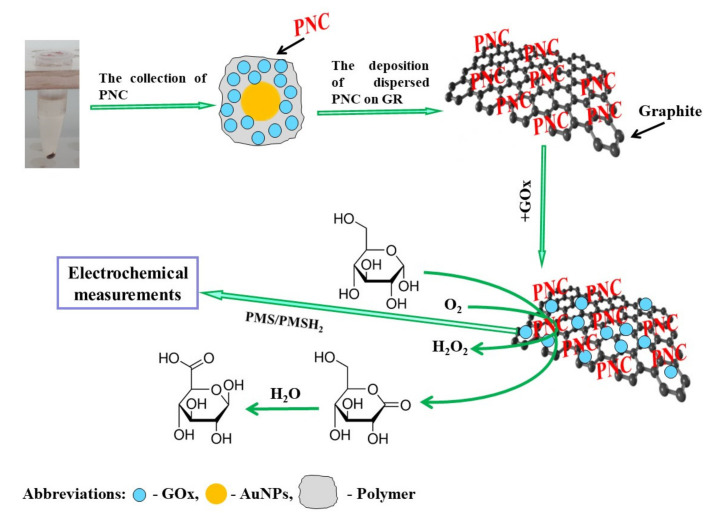
Schematic representation of the deposition of PANI-AuNPs_(6 nm)_-GOx or Ppy-AuNPs_(6 nm)_-GOx nanocomposites (PNC) on the surface of GR electrode with the following additional enrichment by GOx, and glucose determination by the developed enzymatic biosensors.

The principles of PANI-AuNPs_(6 nm)_-GOx or Ppy-AuNPs_(6 nm)_-GOx nanocomposites synthesis, their deposition on the surface of GR electrode with the following additional enrichment by GOx and electrochemical investigations are presented in Figure 1. In the presence of glucose and dissolved oxygen, GOx is able to generate gluconolactone and hydrogen peroxide, which is necessary to initiate monomer’s polymerization reaction [35]. PMS is able to oxidize the reduced redox site (FADH_2_) of GOx. Later, electrons from the reduced PMSH_2_ are transferred directly to the positively charged surface of GR electrode and/or through AuNPs present on the GR electrode [38].

## 3. Results and Discussion

### 3.1. Characterization of Biosensors Based on GR/PANI-GOx/GOx, GR/Ppy-GOx/GOx, GR/PANI-AuNPs_(6 nm)_-GOx/GOx or GR/Ppy-AuNPs_(6 nm)_-GOx/GOx Electrodes by Cyclic Voltammetry

**Figure 2:**
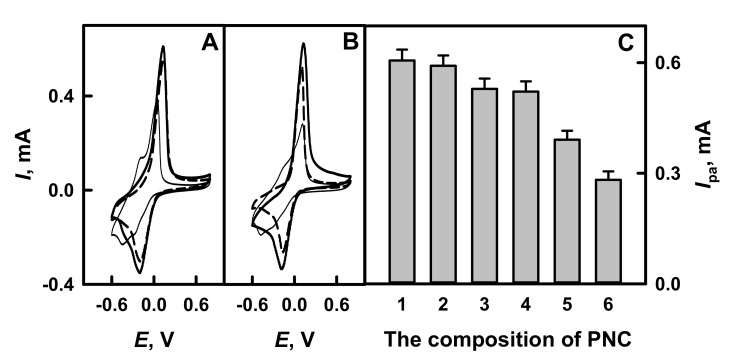
The cyclic voltammograms of GR electrode modified by PANI-GOx/GOx, PANI-AuNPs_(6 nm)_-GOx/GOx or PANI-AuNPs_(6 nm)_-GOx (**A**), Ppy-GOx/GOx, Ppy-AuNPs_(6 nm)_-GOx/GOx or Ppy-AuNPs_(6 nm)_-GOx (**B**) and the diagrams of glucose biosensors based on GR electrodes modified by different composition of polymer nanocomposites (**C**). Conditions: (**A**,**B**) GR/PANI-GOx/GOx and GR/Ppy-GOx/GOx (dashed line), GR/PANI-AuNPs_(6 nm)_-GOx/GOx and GR/Ppy-AuNPs_(6 nm)_-GOx/GOx (solid line), GR/PANI-AuNPs_(6 nm)_-GOx and GR/Ppy-AuNPs_(6 nm)_-GOx (thin line); (**C**) GR/Ppy-AuNPs_(6 nm)_-GOx/GOx (1 column), GR/PANI-AuNPs_(6 nm)_-GOx/GOx (2 column), GR/PANI-GOx/GOx (3 column), GR/Ppy-GOx/GOx (4 column), GR/PANI-AuNPs_(6 nm)_-GOx (5 column) and GR/Ppy-AuNPs_(6 nm)_-GOx (6 column). Analytical signals were registered in 0.05 mol L^−1^ SA buffer, pH 6.0, with 0.01 mol L^−1^ KCl and 6.0 mmol L^−1^ PMS by cyclic voltammetry.

The form of cyclic votammograms and peak shifts are providing important information on the action mechanism and charge transfer of the electrochemical species [34]. The electrochemical behavior of glucose present on GR electrode, which is modified by PANI-GOx/GOx, Ppy-GOx/GOx, PANI-AuNPs_(6 nm)_-GOx/GOx or Ppy-AuNPs_(6 nm)_-GOx/GOx nanocomposites, was investigated in 0.05 mol L^−1^ SA buffer, pH 6.0, with 0.01 mol L^−1^ KCl in the presence of 6 mmol L^−1^ PMS by cyclic voltammetry in potential diapason from −0.60 to +0.80 V. To evaluate the influence of additionally on nanocomposite added GOx, the cyclic voltamperograms of GR/PANI-AuNPs_(6 nm)_-GOx and GR/Ppy-AuNPs_(6 nm)_-GOx were registered. As it is seen from Figure 2A,B, the redox reactions obtained on CR electrodes modified by polymer nanocomposites are reversible and are characterized by well-defined anodic and cathodic peaks. The potential of anodic peaks (*E*_pa_) of GR electrodes modified by PANI-GOx/GOx, Ppy-GOx/GOx, PANI-AuNPs_(6 nm)_-GOx/GOx or Ppy-AuNPs_(6 nm)_-GOx/GOx composites was determined at +0.12 V vs. Ag/AgCl_(3 mol L_^−1^_KCl)_. The potential of cathodic peaks (*E*_pc_) of GR electrode modified by PANI-GOx/GOx or Ppy-GOx/GOx was −0.14 V, while for PANI-AuNPs_(6 nm)_-GOx/GOx and Ppy-AuNPs_(6 nm)_-GOx/GOx it was −0.17 and −0.15 V, respectively. Diffusion-controlled, reversible electrochemical process was observed: the peak separation (Δ*E*_p_ *= E*_pa_*-E*_pc_) for PANI-GOx/GOx and Ppy-GOx/GOx was 0.26 V, while for PANI-AuNPs_(6 nm)_-GOx/GOx and Ppy-AuNPs_(6 nm)_-GOx/GOx it was 0.29 and 0.27 V, respectively. This fact indicates that the reversibility was of high degree and both (PANI-AuNPs_(6 nm)_-GOx and Ppy-AuNPs_(6 nm)_-GOx) polymer nanocomposites increased the efficiency of charge transfer between GOx and GR.

It was tentatively determined that glucose biosensors based on GR electrodes modified by GOx-containing nanocomposites are sensitive towards glucose. As is seen from Figure 2C, the presence of AuNPs_(6 nm)_ in polyaniline- and polypyrrole-based nanocomposites (PANI-AuNPs_(6 nm)_-GOx/GOx and Ppy-AuNPs_(6 nm)_-GOx/GOx) increased anodic current by 1.12 and 1.16 times, respectively, if compared with that registered for electrodes modified by PANI-GOx/GOx and Ppy-GOx/GOx. This fact indicates that AuNPs show good electrocatalytic activity towards electrochemical oxidation of glucose. The addition of GOx after electrode modification by polymer nanocomposites is recommended to increase the current response by 1.51 and 2.15 times for GR/PANI-AuNPs_(6 nm)_-GOx/GOx and GR/Ppy-AuNPs_(6 nm)_-GOx/GOx electrodes, in the comparison with results obtained using GR/PANI-AuNPs_(6 nm)_-GOx and GR/Ppy-AuNPs_(6 nm)_-GOx electrodes.

### 3.2. The Influence of Polymer Nanocomposites Composition and Layer Thickness on the Current Response Registered by Amperometry

The composition of polymer nanocomposites has a significant influence on the sensitivity and analytical characteristics of the here developed amperometric biosensors. Thoroughly, the influence of the composition of polymer-GOx nanocomposites with glucose oxidase without and with AuNPs_(6 nm)_ was investigated in this chapter. To determine the advantages of polymer-GOx nanocomposites, the measurements were performed using GR electrodes modified by polymer nanocomposites of different composition: in the presence of GOx without and with AuNPs_(6 nm)_. The investigations were performed in SA buffer, pH 6.0, with 0.01 mol L^−1^ KCl and 6.0 mmol L^−1^ PMS.

**Figure 3:**
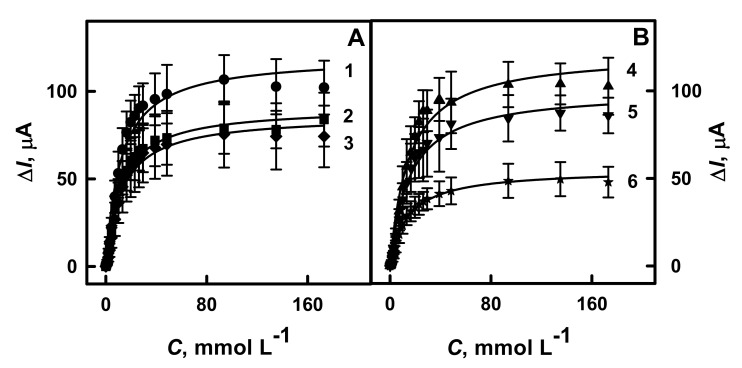
Calibration plots of glucose biosensors based on GR electrodes modified by polymer nanocomposites with the following additional enrichment by GOx: (**A**) PANI-AuNPs_(6 nm)_-GOx/GOx or PANI-GOx/GOx and (**B**) Ppy-AuNPs_(6 nm)_-GOx/GOx and Ppy-GOx/GOx. Conditions: GR electrodes covered by PANI-AuNPs_(6 nm)_-GOx/GOx (1 line), PANI-GOx/GOx (2 line), Ppy-AuNPs_(6 nm)_-GOx/GOx (4 line) and Ppy-GOx/GOx (5 line). For comparison, GR electrodes modified with AuNPs_(6 nm)_-GOx (3 line) and GOx (6 line) were investigated. Amperometric responses were registered in 0.05 mol L^−1^ SA buffer, pH 6.0, with 0.01 mol L^−1^ KCl and 6.0 mmol L^−1^ PMS.

**Figure 4:**
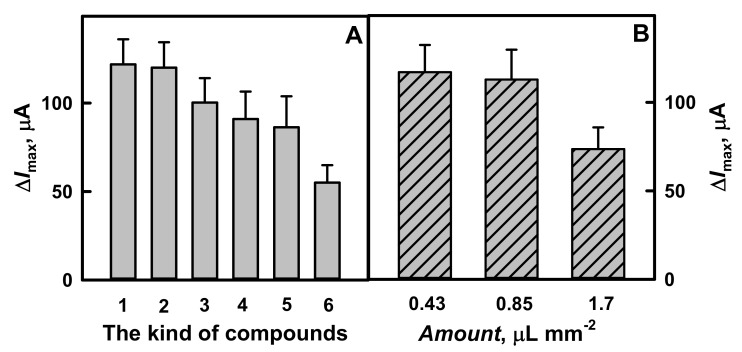
The diagrams of amperometric current response registered using GR electrodes (surface area 0.071 cm^2^) modified by: (**A**) different kind of compounds and (**B**) different amount of PANI-AuNPs_(6 nm)_-GOx nanocomposites. Conditions: (**A**) GR/Ppy-AuNPs_(6 nm)_-GOx/GOx (1 column), GR/PANI-AuNPs_(6 nm)_-GOx/GOx (2 column), GR/Ppy-GOx/GOx (3 column), GR/PANI-GOx/GOx (4 column) electrodes; (**B**) GR/PANI-AuNPs_(6 nm)_-GOx/GOx electrode. For comparison, GR/AuNPs_(6 nm)_-GOx (5 column) and GR/GOx (6 column) electrodes were used. Amperometric responses were registered in 0.05 mol L^−1^ SA buffer, pH 6.0, with 0.01 mol L^−1^ KCl and 6.0 mmol L^−1^ PMS.

The hyperbolic dependences of the current response registered by amperometry on the concentration of glucose in the diapason increased from 0.10 to 173 mmol L^−1^, and the influence of these kinds of compounds on the analytical response are presented in Figure 3 and Figure 4A, respectively. As is seen from Figure 3, the registered hyperbolic dependences Δ*I* vs. glucose concentration were in agreement with the Michaelis–Menten kinetics. The difference of the maximal current response generated during the enzymatic reaction and the apparent Michaelis constant were correspondingly *a* and *b* parameters of the hyperbolic function (*y* = *ax/(b* + *x)*) and were used for the approximation of the obtained results.

As is seen from Figure 3 and Figure 4A, glucose biosensors based on GR electrode modified by polymer nanocomposites with GOx and AuNPs_(6 nm)_ after additional enrichment by GOx are characterized by significantly higher sensitivity. The maximal current was higher for nanocomposites containing AuNPs_(6 nm)_. Particularly for GR/Ppy/AuNPs_(6 nm)_-GOx/GOx (Δ*I*_max_ = 122 μA) and GR/PANI/AuNPs_(6 nm)_-GOx/GOx (Δ*I*_max_ = 120 μA) the current response increased by 1.22 and 1.32 times, respectively, if compared with results obtained using electrode modified by Ppy-GOx/GOx (Δ*I*_max_ = 100 μA) or PANI-GOx/GOx (Δ*I*_max_ = 91.0 μA). These results are in good agreement with that presented in previous investigations by cyclic voltammetry. Obtained results present the advantage of biosensors based on polymer nanocomposites, which contain AuNPs_(6 nm)_ (Figure 4A). These results illustrate that Δ*I*_max_ for GR electrodes modified by PANI-AuNPs_(6 nm)_-GOx/GOx or Ppy-AuNPs_(6 nm)_-GOx/GOx has increased by 1.39 and 1.41 times in the comparison with that registered by GR electrode modified only with AuNPs_(6 nm)_-GOx (Δ*I*_max_ = 86.3 μA). The value of Δ*I*_max_ determined for GR electrodes modified by PANI-GOx/GOx or Ppy-GOx/GOx was 1.65 and 1.81 times higher if compared with that determined for the electrode modified only by GOx (Δ*I*_max_ = 55.1 μA).

High values of the apparent Michaelis constant determined for electrodes based on PANI-AuNPs_(6 nm)_-GOx/GOx and Ppy-AuNPs_(6 nm)_-GOx/GOx show an increase linear detection range of glucose, which is the main advantage when the determination is performed directly in real samples. In our investigations, GR/PANI-AuNPs_(6 nm)_-GOx/GOx, GR/Ppy-AuNPs_(6 nm)_-GOx/GOx, GR/PANI-GOx/GOx or GR/Ppy-GOx/GOx electrodes were characterized by *K*_M_ value of 15.1, 11.7, 14.9 and 11.8 mmol L^−1^, respectively. According to another research, this effect indicates a strong affinity between the enzyme and glucose [19,31]. Therefore, PANI-AuNPs_(6 nm)_-GOx/GOx and Ppy-AuNPs_(6 nm)_-GOx/GOx were the most efficient and were used in further investigations.

The sensitivity of the designed biosensors depend on the composition of the polymer layer deposited on the surface of electrode. The influence of the thickness of polymer nanocomposites, which were deposited on GR electrodes, on the current response of the biosensor was investigated when different amounts of PANI-AuNPs_(6 nm)_-GOx nanocomposites were equally distributed on GR electrode surface and the same amount of GOx was additionally immobilized over formed nanocomposite layer. As it is seen from the diagrams presented in Figure 4B, the difference of the maximal current response has decreased in the sequence—120, 119 and 76.9 μA, with an increase of the amount of PANI-AuNPs_(6 nm)_-GOx nanocomposites of 0.43, 0.85 to 1.7 μL mm^−2^, respectively.

The value of Δ*I*_max_ registered by GR electrodes modified by 0.43 μL mm^−2^ polymer nanocomposites was similar (only 1.01 times higher) to 0.85 μL mm^−2^ and 1.56 times higher than that obtained using 1.7 μL mm^−2^ polymer nanocomposites. The decrease of Δ*I*_max_ of the developed biosensor can be explained by the formation of a thick layer of PANI-AuNPs_(6 nm)_-GOx nanocomposites on GR electrode, which limits the diffusion of glucose to the surface of electrode. The lower amount of polymer nanocomposites than that of 0.43 μL mm^−2^ was not used in our system through similarity of the difference of maximal current for GR electrode modified by 0.85 or 1.7 μL mm^−2^ of polymer nanocomposites. In this way, the following investigations were performed in the presence of 0.43 μL mm^−2^ of polymer nanocomposites.

### 3.3. The Evaluation of the Analytical Characteristics of GR Electrodes Modified by PANI-AuNPs_(6 nm)_-GOx/GOx, Ppy-AuNPs_(6 nm)_-GOx/GOx, PANI-GOx/GOx or Ppy-GOx/GOx

**Figure 5:**
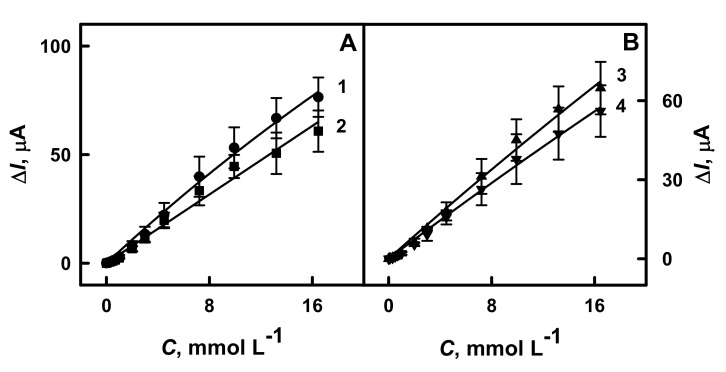
The linear detection range of biosensors based on GR electrodes modified by: (**A**) PANI-AuNPs_(6 nm)_-GOx/GOx (1 line) and PANI-GOx/GOx (2 line), (**B**) Ppy-AuNPs_(6 nm)_-GOx/GOx (3 line) and Ppy-GOx/GOx (4 line). Conditions: amperometric responses were registered in 0.05 mol L^−1^ SA buffer, pH 6.0, with 0.01 mol L^−1^ KCl and 6.0 mmol L^−1^ PMS.

The next step of the research was dedicated to the evaluation of the analytical characteristics (linear range of glucose determination, correlation coefficient, sensitivity) and stability of the developed analytical systems. Firstly, the linear detection range of glucose determination was measured using GR electrode modified by PANI-AuNPs_(6 nm)_-GOx/GOx, Ppy-AuNPs_(6 nm)_-GOx/GOx, PANI-GOx/GOx or Ppy-GOx/GOx. As it is seen from the obtained calibration curves (Figure 5), the linear detection range of glucose for all investigated systems was up to 16.5 mmol L^−1^. The achieved linear detection range in our study was wider than for glucose biosensors based on carbon-ink/GOx modified stainless steel electrode (linear range up to 1 mmol L^−1^) [19], glassy carbon (GC) electrode modified by graphene/PANI/AuNPs/GOx (linear range up to 1.12 mmol L^−1^) [37] or graphene/nano-Au/GOx (linear range up to 3 mmol L^−1^) [24]. It should be mentioned that only the linear range up to 0.7 mmol L^−1^ was registered using the GR electrode modified by the long-chain Ppy/AuNPs_(AuCl4_^-^_)_-GOx nanocomposite [39]; hence, in our present research we successfully improved the performance of the biosensor applying dispersed short-chain conducting polymer nanocomposite with an additional layer of GOx. Additionally, the linear ranges for GR/PANI-AuNPs_(6 nm)_-GOx/GOx, GR/Ppy-AuNPs_(6 nm)_-GOx/GOx, GR/PANI-GOx/GOx and GR/Ppy-GOx/GOx were characterized by the correlation coefficient of 0.9968, 0.9961, 0.9927 and 0.9977, respectively (Table 1). As it is seen from Figure 5, the linear detection range of glucose is without the intercept on *x*- or *y*-axis that indicates the suitability of the developed biosensors for analyte biosensing in biological samples.

The sensitivity of the developed biosensors based on GR electrodes modified by PANI-AuNPs_(6 nm)_-GOx/GOx, Ppy-AuNPs_(6 nm)_-GOx/GOx, PANI-GOx/GOx or Ppy-GOx/GOx towards 16.5 mmol L^−1^ of glucose was determined as 65.4, 55.4, 52.0 and 48.0 μA mM^−1^ cm^−2^, respectively (Table 1). The presence of AuNPs_(6 nm)_ in the polymerization solution and in PANI-AuNPs_(6 nm)_-GOx and Ppy-AuNPs_(6 nm)_-GOx nanocomposites increased the sensitivity of glucose biosensor based on GR/PANI-AuNPs_(6 nm)_-GOx/GOx or GR/Ppy-AuNPs_(6 nm)_-GOx/GOx by 1.26 and 1.15 times, respectively, if compared with results obtained for GR/PANI-GOx/GOx and GR/Ppy-GOx/GOx electrodes. Glucose biosensors based on GR electrodes modified by PANI-AuNPs_(6 nm)_-GOx/GOx or Ppy-AuNPs_(6 nm)_-GOx/GOx are 5.61 and 4.76 times more sensitive than glucose biosensor based on GC/PLL/RGO-ZrO_2_ electrode (11.65 μA mM^−1^ cm^−2^) [23]. The sensitivity of the developed glucose biosensors based on GR/PANI-AuNPs_(6 nm)_-GOx/GOx or GR/Ppy-AuNPs_(6 nm)_-GOx/GOx due to the use of another electrochemical method was very similar or a little bit smaller than that obtained in our previous study in the presence of long-chain Ppy/AuNPs_(AuCl4_^-^_)_-GOx nanocomposite (77.2 μA mM^−1^ cm^−2^) [39]. The sensitivity of GR/Ppy-AuNPs_(6 nm)_-GOx/GOx-based glucose biosensor developed in recent research is 2.55 times higher than the glucose biosensor based on AuNP_(13nm)_ electrochemically deposited on GR electrode and modified by GOx and Ppy (21.7 μA mM^−1^ cm^−2^) [38]. Moreover, the sensitivity of the developed GR/Ppy-AuNPs_(6 nm)_-GOx/GOx-based glucose biosensor is 18 times higher if compared with the glucose biosensor based on GC electrode modified by OOPpy_(300 s)_-nanoAu and GOx (3.07 μA mM^−1^ cm^−2^) [29].

The repeatability of biosensors based on GR electrodes modified by PANI-AuNPs_(6 nm)_-GOx/GOx, Ppy-AuNPs_(6 nm)_-GOx/GOx, PANI-GOx/GOx or Ppy-GOx/GOx GR was investigated in the presence of 2.99 mmol L^−1^ of glucose. The relative standard deviations (RSD) calculated for these biosensors were 4.67%, 5.89%, 6.10% and 9.72%, respectively. Developed glucose biosensors based on GR/PANI-AuNPs_(6 nm)_-GOx/GOx or GR/Ppy-AuNPs_(6 nm)_-GOx/GOx exhibited better repeatability (up to 3.0 and 2.4 times) than that obtained previously in the presence of long-chain polymer nanocomposites (for Ppy/AuNPs_(AuCl4_^-^_)_-GOx RSD was 13.9%) [39]. The analytical response obtained using the developed in this paper glucose biosensor was two times faster than that obtained in our previous paper where long-chain polymer nanocomposites were used (20 s) [39]. As is seen from Table 1, the limit of detection for GR/PANI-AuNPs_(6 nm)_-GOx/GOx and GR/Ppy-AuNPs_(6 nm)_-GOx/GOx electrodes was almost similar — 0.070 and 0.071 mmol L^−1^, respectively. Higher values of LOD for biosensors were determined using GR/PANI-GOx/GOx (0.084 mmol L^−1^) or GR/Ppy-GOx/GOx (0.10 mmol L^−1^) electrodes. Biosensors based on GR/PANI-AuNPs_(6 nm)_-GOx/GOx and GR/Ppy-AuNPs_(6 nm)_-GOx/GOx electrodes are characterized by lower LOD if compared with GR/PANI-GOx/GOx and GR/Ppy-GOx/GOx electrodes. The limit of detection determined for the developed biosensor based on short-chain PANI-AuNPs_(6 nm)_-GOx composites with following immobilization of GOx was 1.4 times lower than that for long-chain Ppy/AuNPs_(AuCl4_^-^_)_-GOx (0.1 mmol L^−1^) nanocomposite [39]. The LOD for biosensor based on Ppy-AuNPs_(6 nm)_-GOx/GOx electrode was 2.82 times lower if compared with previously developed biosensor based on electrochemically deposited AuNPs_(13nm)_, immobilized GOx and covered by Ppy (LOD 0.20 mmol L^−1^) [38].

### 3.4. The Stability of Glucose Biosensors Based on GR Electrodes Modified by PANI-AuNPs_(6 nm)_-GOx/GOx or Ppy-AuNPs_(6 nm)_-GOx/GOx

**Figure 6:**
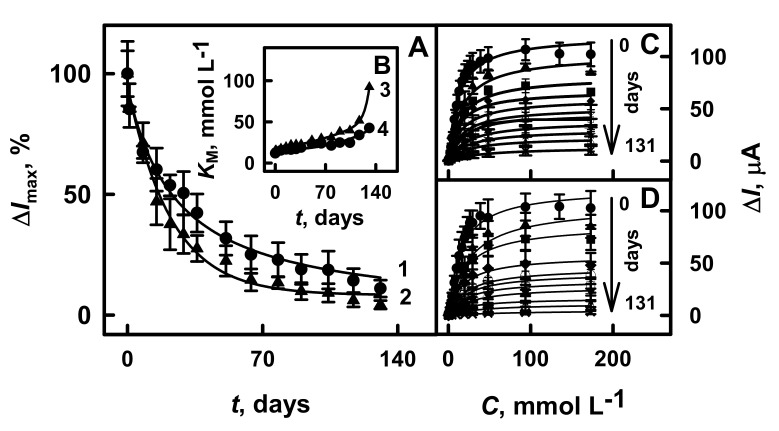
The changes of analytical signal (**A**), and the increase of the apparent Michaelis constant over time (**B**), calibration plots of glucose biosensors based on GR/PANI-AuNPs_(6 nm)_-GOx/GOx electrode (**C**) and on GR/Ppy-AuNPs_(6 nm)_-GOx/GOx electrode (**D**). Details of the presented calibration plots: GR/PANI-AuNPs_(6 nm)_-GOx/GOx electrode ((**A**) line 1, (**B**) line 4, and (**C**) all lines) and GR/Ppy-AuNPs_(6 nm)_-GOx/GOx electrode ((**A**) line 2, (**B**) line 3 and (**D**) all lines). Amperometric responses were registered in 0.05 mol L^−1^ SA buffer, pH 6.0, with 0.01 mol L^−1^ KCl and 6.0 mmol L^−1^ PMS.

For the stability test, GR electrodes modified by PANI-AuNPs_(6 nm)_-GOx/GOx or Ppy-AuNPs_(6 nm)_-GOx/GOx were stored at +4 °C in a closed vessel hanging over SA buffer solution between electrochemical measurements for 131 days. The changes of analytical signal over time and the apparent Michaelis constant for the developed glucose biosensors are presented in Figure 6A,B, respectively.

As is seen from Figure 6C,D, the hyperbolic dependences of current responses on glucose concentration were in agreement with Michaelis–Menten kinetics. The analytical signal towards glucose for biosensors based on GR/PANI-AuNPs_(6 nm)_-GOx/GOx and GR/Ppy-AuNPs_(6 nm)_-GOx/GOx electrodes considerably decreased during first 8 days, and 67.4% and 71.4% of initially current response was registered (Figure 6A). The decrease of current response might be explained by a low stability of the enzyme over time due to their denaturation and deactivation [4,6]. From 51 to 131 days, the current response of GR/PANI-AuNPs_(6 nm)_-GOx/GOx and GR/Ppy-AuNPs_(6 nm)_-GOx/GOx electrodes towards glucose decreased from 31.8% down to 11.0% and from 24.4% down to 4.28%, respectively. The *τ*_1/2_ values, which represent the time after that 50% of the initial current response is registered for glucose biosensors based on GR/PANI-AuNPs_(6 nm)_-GOx/GOx or GR/Ppy-AuNPs_(6 nm)_-GOx/GOx electrodes, were determined as 22 days and 17 days, respectively. These results illustrate that the biosensor based on GR/PANI/AuNPs_(6 nm)_-GOx/GOx electrode is 1.29 times more stable than that based on the GR/Ppy/AuNPs_(6 nm)_-GOx/GOx electrode.

The developed glucose biosensor based on the GR electrode modified by PANI-AuNPs_(6 nm)_-GOx/GOx was 3.14 times more stable than that of the Pt/PANI/gold nanorod composites/GOx electrode without mediator (*τ*_1/2_ was determined as 7.0 days) [44]. The glucose biosensor based on the GR/Ppy-AuNPs_(6 nm)_-GOx/GOx electrode was 1.73 times more stable than that previously reported for biosensors based on the GR electrode with electrochemical deposited AuNPs_(13nm)_, immobilized GOx and modified by Ppy (9.8 days of *τ*_1/2_) [38]. In this paper, we developed a biosensor that is based on short-chain PANI-AuNPs_(6 nm)_-GOx with a following immobilization of GOx that was by 17, 6.7 and 1.2 times more stable than the biosensor based on long-chain PANI/AuNPs_(6 nm)_-GOx (*τ*_1/2_ = 1.3 days), Ppy/AuNPs_(6 nm)_-GOx (*τ*_1/2_ = 3.3 days) and Ppy/AuNPs_(AuCl4_^-^_)_-GOx (*τ*_1/2_ = 19 days) [39]. The higher stability of the biosensor is very important advantage.

The *K*_M_ constant increased from 15.4 to 92.3 mmol L^−1^ for the GR/PANI-AuNPs_(6 nm)_-GOx/GOx electrode and from 12.9 to 42.4 mmol L^−1^ for the GR/Ppy-AuNPs_(6 nm)_-GOx/GOx electrode within the time frame ranging from 1 to 131 days (Figure 6B). It is seen that the value of the *K*_M_ for the GR electrode modified by Ppy-AuNPs_(6 nm)_-GOx/GOx after 131 days of electrode’s storage stability was 2.18 times better than that determined for electrodes modified by PANI-AuNPs_(6 nm)_-GOx/GOx. However, the higher value of *K*_M_ correlates with the decreased sensitivity of the biosensor. The glucose biosensor based on GR/PANI-AuNPs_(6 nm)_-GOx/GOx was selected for further investigation due to their rather good performance (high sensitivity and good stability, low LOD and good reproducibility (5.8% for 11 measurements) (Figure S1 in the Supplementary Materials)). Additionally, this biosensor was applied for glucose determination in the serum sample.

### 3.5. The Application of Biosensor Based on GR/PANI-AuNPs_(6 nm)_-GOx/GOx for Determination of Glucose in Serum Samples

Glucose is considered as the most important sugar, which is always present in human’s blood serum [21,45]. Other sugars such as fructose, mannose, galactose, xylose, and saccharose are considered stereoisomers of glucose and are characterized by the same molecular formula and sequence of bonded atoms, similar to the molecule of glucose [46]. Both AA and UA are antioxidants that are interfering during glucose biosensing through their influence on the response of the biosensor [44,45,46,47,48,49]. UA in a combination with another oxidant is able to generate radicals, which can promote various side reactions and damage of cells [48]. Meanwhile, the carboxylate group in polymer structures is able to decrease the penetration rate of negatively charged interfering compounds towards the electrode [44]. Therefore, such groups can increase the selectivity of sensors, which are used for the determination of glucose in samples that contain these compounds.

**Figure 7:**
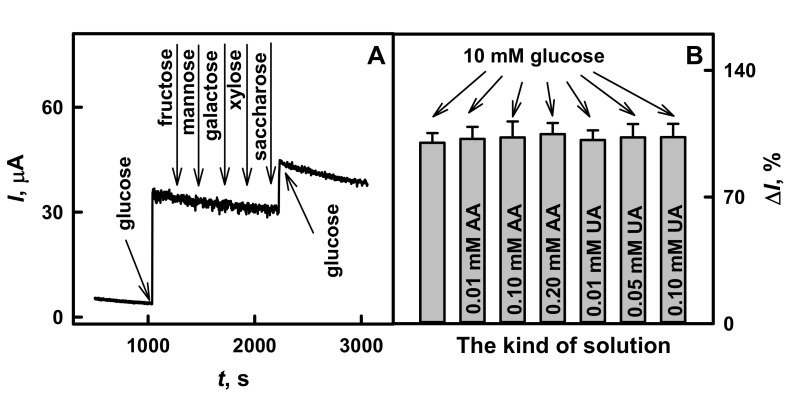
The influence of interfering species on the current response of glucose biosensor based on the GR/PANI-AuNPs_(6 nm)_-GOx/GOx electrode. Conditions: (**A**) amperogram registered in 10× diluted serum sample after the addition of 10.0 mmol L^−1^ glucose and 1.00 mmol L^−1^ of fructose, mannose, galactose, xylose or saccharose, (**B**) the diagram of registered current response in 10× diluted serum sample after the addition of 10.0 mmol L^−1^ glucose (first grey column without marking inside); of 10.0 mmol L^−1^ glucose with 0.01, 0.10 or 0.20 mmol L^−1^ of AA; of 10.0 mmol L^−1^ glucose with 0.01, 0.05 or 0.10 mmol L^−1^ of UA. Amperometric responses were registered in a 10× diluted sample of serum in the presence of 6 mmol L^−1^ PMS.

The selectivity tests of the glucose biosensor based on the GR/PANI-AuNPs_(6 nm)_-GOx/GOx electrode to interfering and electroactive species were performed in 10× diluted serum samples. To evaluate the influence of sugars on the current response of the developed biosensor, the serum containing 10.0 mmol L^−1^ of glucose with the following addition of 1.00 mmol L^−1^ fructose, mannose, galactose, xylose and saccharose was tested (Figure 7A). As is seen from the obtained results, the developed glucose biosensor based on GR/PANI-AuNPs_(6 nm)_-GOx/GOx electrode was sensitive only to glucose.

The influence of such electroactive species as AA and UA on the registered current response was investigated. The assessed concentrations of AA and UA were higher than their normal physiological concentrations: 0.141 mmol L^−1^ for AA [43,50] and 0.1 mmol L^−1^ for UA [47,51]. The results presented in Figure 7B illustrate that the addition of electroactive species has a moderate influence on the current response of the here evaluated glucose biosensor based on the GR/PANI-AuNPs_(6 nm)_-GOx/GOx electrode. The addition of 10.0 mmol L^−1^ glucose with 0.01 and 0.10 mmol L^−1^ of AA increased the current response by 2.11% and 2.89%, while the addition of 10.0 mmol L^−1^ glucose with 0.20 mmol L^−1^ of AA increased the current response by 4.74% if compared with current response after the addition of 10.0 mmol L^−1^ glucose without any AA. The presence of 0.01, 0.05 and 0.10 mmol L^−1^ UA in the solution of 10.0 mmol L^−1^ glucose increased the current response of the developed biosensor by 1.49%, 2.99% and 3.08%, respectively, in comparison with the results obtained after the addition of 10.0 mmol L^−1^ glucose without any UA. The anti-interference capability of the developed glucose biosensor was higher than that described by other authors for the glucose biosensor based on GC electrode modified by graphene/nano-Au/GOx nanocomposites (the interference of 5.6% for AA and of 3.2% for UA was registered) [24]. If the obtained characteristics are compared with the previously published biosensor based on the long-chain Ppy/AuNPs_(AuCl4_^-^_)_-GOx nanocomposites [39], it was shown that the developed glucose biosensor is 1.53 and 2.13 times more resistant to 0.01 or 0.10 mmol L^−1^ of AA, and 1.47 and 4.48 times more resistant to 0.01 or 0.05 mmol L^−1^ UA. Just the insignificant interference of electroactive species indicates that the developed glucose biosensor based on GR/PANI-AuNPs_(6 nm)_-GOx/GOx electrode is characterized by very good anti-interference ability towards the here evaluated interfering materials.

Glucose concentration of nondiabetic human’s blood serum usually is in the range from 3 to 8 mmol L^−1^ [29,51,52], while for diabetic human‘s blood serum glucose concentration might be up to 30 mmol L^−1^ [3,11,29]. The suitability of the designed biosensor based on the GR electrode modified by PANI-AuNPs_(6 nm)_-GOx/GOx for glucose determination in the diluted serum sample was assessed using the addition method. The results of the determined glucose concentration and the recovery are presented in Table 2. It was determined that the recovery ratios in this paper improved the enzymatic glucose biosensor in the serum samples from 95.0% to 98.5%, which are similar to the results obtained using GC/OOPpy_(300 s)_-nanoAu/GOx electrode (96%) [29], and are better than the results obtained using the GR electrode directly modified by long-chain Ppy/AuNPs_(AuCl4_^-^_)_-GOx nanocomposites (92.7–94.8%) [39].

The analytical characteristics of glucose biosensor based on dispersed enzymatically synthesized PANI nanocomposites with following immobilization of GOx were improved in comparison to polymeric nanocomposites-based nanoparticles [39]. It should be noted that the advantages of the developed biosensor are: (i) high sensitivity (65.4 μA mM^−1^ cm^−2^) and stability (*τ*_1/2_ = 22 days), (ii) low limit of detection (0.070 mmol L^−1^), good reproducibility (5.8%) and repeatability (4.67%), (iii) wide linear range (up to 16.5 mmol L^−1^) and small amount of aliquot required for the analysis, (iv) multiple use and rather low price of single analysis, (v) fast analytical response (10 s), high resistance to interfering species (10.0 mmol L^−1^ glucose with 0.01, 0.10 or 0.20 mmol L^−1^ of AA increased the current response by 2.11, 2.89 or 4.74%; 10.0 mmol L^−1^ glucose with 0.01, 0.05 and 0.10 mmol L^−1^ UA increased the current response by 1.49, 2.99 and 3.08%) and suitability for glucose determination (95.0–98.5%). Thus, these characteristics allow the application of the developed biosensor for the determination of glucose in real samples.

## 4. Conclusions

In this research, we demonstrated the improved properties and analytical parameters of a glucose biosensor developed using dispersed nanocomposites based on GOx and AuNPs_(6 nm)_ embedded within the conducting polymers PANI or Ppy. Biosensors with shorter polymeric chains are characterized by good reversibility of the soluble redox mediator. Our results show that the glucose biosensors based on GR/PANI-AuNPs_(6 nm)_-GOx/GOx or GR/Ppy-AuNPs_(6 nm)_-GOx/GOx electrodes are characterized by high sensitivity, low limit of detection, wide linear range, good repeatability, sufficient storage stability. Additionally, the glucose biosensor based on GR/PANI-AuNPs_(6 nm)_-GOx/GOx electrode operates well in the serum sample and detects glucose in the presence of interfering species. This glucose biosensor enables the reduction of the amount of aliquot, which is required for the determination of the analyte. The methodology presented in this paper could be used for the design and fabrication of improved enzymatic biosensors for biomedical analysis. Further modification of GR/PANI-AuNPs_(6 nm)_-GOx/GOx or GR/Ppy-AuNPs_(6 nm)_-GOx/GOx electrodes by insoluble redox mediator will be the next step in this our research direction.

## Figures and Tables

**Table 1:** The comparison of analytical characteristics of glucose biosensors based on GR/PANI-AuNPs_(6 nm)_-GOx/GOx, GR/Ppy-AuNPs_(6 nm)_-GOx/GOx, GR/PANI-GOx/GOx or GR/Ppy-GOx/GOx. All experimental conditions were the same as reported in Figure 5.

The Composition of Polymer Nanocomposites on GR	LOD, mmol L^−1^	Sensitivity, μA mM^−1^ cm^−2^	Linear Detection, mmol L^−1^	Correlation Coefficient
PANI-AuNPs_(6 nm)_-GOx/GOx	0.070	65.4	0.10–16.5	0.9968
Ppy-AuNPs_(6 nm)_-GOx/GOx	0.071	55.4	0.10–16.5	0.9961
PANI-GOx/GOx	0.084	52.0	0.10–16.5	0.9936
Ppy-GOx/GOx	0.10	48.0	0.10–16.5	0.9977

**Table 2:** The determination and recovery ratio of glucose in the serum sample were investigated by biosensors based on the GR/PANI-AuNPs_(6 nm)_-GOx/GOx electrode (*n*—number of measurements, RSD—repeatability).

Added Concentration, mmol L^−1^	Detected Concentration, mmol L^−1^	RSD, %	Recovery Ratio, %
0.10	0.095 (4)	8.13	95.0
0.30	0.291 (4)	7.80	97.0
0.50	0.490 (4)	4.99	98.0
0.70	0.676 (4)	4.31	96.6
1.00	0.972 (4)	5.42	97.2
2.00	1.92 (5)	7.42	96.0
4.48	4.40 (4)	7.09	98.2
7.21	7.10 (4)	7.67	98.5

Amperometric responses were measured in 10× diluted serum samples in the presence of 6 mmol L^−1^ PMS.

## Data Availability

Not applicable.

## Supplementary Materials

The following are available online at https://www.mdpi.com/article/10.3390/polym13132173/s1, Figure S1: The reproducibility of enzymatic biosensors based on GR electrode modified by PANI-AuNPs_(6 nm)_-GOx/GOx for 19.7 mmol L^−1^ of glucose concentration. Amperometric responses were registered in 0.05 mol L^−1^ SA buffer, pH 6.0, with 0.01 mol L^−1^ KCl and 6.0 mmol L^−1^ PMS.
