# Supplementary material for: Dispersed Conducting Polymer Nanocomposites with Glucose Oxidase and Gold Nanoparticles for the Design of Enzymatic Glucose Biosensors
Source: Polymers (Basel). 2021 Jun 30;13(13):2173. doi: 10.3390/polym13132173 (PMC8271668; doi:10.3390/polym13132173)
Supplement: Supplementary file 1 [file polymers-13-02173-s001.zip › polymers-1277633-supplementary.pdf]

Supplementary material

Dispersed Conducting Polymer Nanocomposites with Glucose Oxidase and Gold Nanoparticles for the Design of Enzymatic Glucose Biosensors

Natalija German [1,2], Almira Ramanaviciene [1,2], Arunas Ramanavicius [3,4]*

[1] Department of Immunology, State Research Institute Centre for Innovative Medicine, Santariskiu 5, LT-08406 Vilnius, Lithuania; natalija.german@imcentras.lt (N.G.); almira.ramanaviciene@chf.vu.lt (AR)

[2] NanoTechnas—Center of Nanotechnology and Materials Science, Faculty of Chemistry and Geosciences, Vilnius University, LT-03225 Vilnius, Lithuania

[3] Department of Physical Chemistry, Faculty of Chemistry and Geosciences, Vilnius University, Naugarduko 24, LT-03225 Vilnius, Lithuania

[4] Division of Materials Science and Electronics, State Scientific Research Institute Center for Physical Sciences and Technology, Savanorių ave. 231, LT-02300 Vilnius, Lithuania

* Correspondence: arunas.ramanavicius@chf.vu.lt

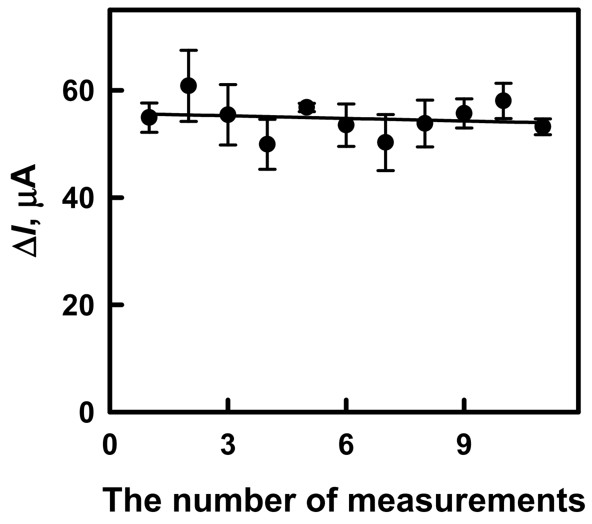

Figure S1. The reproducibility of enzymatic biosensors based on GR electrode modified by PANI-AuNPs$_{(6\ nm)}$-GOx/GOx for 19.7 mmol L^{-1} of glucose concentration. Amperometric responses were registered in 0.05 mol L^{-1} SA buffer, pH 6.0, with 0.01 mol L^{-1} KCl and 6.0 mmol L^{-1} PMS.
